# Association between GHQ-12, Duke-UNC-11, Physical Activity, and Self-Perceived Health in Spanish Adults with Cancerous Tumours: A Cross-Sectional Study

**DOI:** 10.3390/healthcare11020192

**Published:** 2023-01-09

**Authors:** Juan Manuel Franco-García, Ángel Denche-Zamorano, Damián Pereira-Payo, Yeray Rodríguez-Redondo, Jorge Carlos-Vivas, Antonio Castillo-Paredes, Miguel Ángel García-Gordillo, Laura Muñoz-Bermejo

**Affiliations:** 1 Health, Economy, Motricity and Education (HEME) Research Group, Faculty of Sport Sciences, University of Extremadura, 10003 Cáceres, Spain; 2 Promoting a Healthy Society (PHeSO) Research Group, Faculty of Sport Sciences, University of Extremadura, 10003 Cáceres, Spain; 3 Social Impact and Innovation in Health (InHEALTH), University of Extremadura, 06810 Mérida, Spain; 4 Physical Activity for Education, Performance and Health (PAEPH) Research Group, Faculty of Sport Sciences, University of Extremadura, 10003 Cáceres, Spain; 5 Grupo AFySE, Investigación en Actividad Física y Salud Escolar, Escuela de Pedagogía en Educación Física, Facultad de Educación, Universidad de Las Américas, Santiago 8370040, Chile; 6 Faculty of Administration and Business, Universidad Autónoma de Chile, Talca 3467987, Chile

**Keywords:** cancer, exercise, fitness, lifestyle, physical therapy

## Abstract

Background: In Spain, people who have overcome some type of cancer have significantly worse self-perceived health (SPH) and higher rates of depression than people who have never suffered any type of cancer. Objective: to explore the relationships among physical activity levels (PAL), perceived social support (PSS), and SPH in terms of mental health and its dimensions in Spanish adults with cancerous tumours. Methods: A correlational study rooted in the National Health Survey 2017 for adults was carried out, including 627 Spanish residents who reported having malignant tumours. Results: A dependent association was found between PAL and SPH (*p* < 0.001). The mental health mean score decreased as PAL increased for the total sample and for both sexes, separately (*p* < 0.001). Low reverse associations were also observed between PAL and mental health (rho: −0.274; *p* < 0.001), successful coping (rho: −0.239; *p* < 0.001) and self-confidence (rho: −0.264; *p* <0.001). Moreover, PSS weakly and inversely correlates with mental health (r: −0.225; *p* < 0.001), successful coping (r: −0.218; *p* < 0.001) and self-confidence (r: −0.231; *p* < 0.001). A binary logistic model showed that active and very active people presented less threat of poor SPH, as did people with higher PSS (*p* < 0.001). Conclusions: Greater levels of physical activity are associated with larger mean scores in the three dimensions of mental health, perceived social support and self-perceived health in people with cancerous tumours.

## 1. Introduction

Depression constitutes an important problem among medical patients, which unfortunately is often forgotten to be treated [1]. Oncology patients and survivors in particular are not offered any treatment for the depressive or psychiatric disorders that may result from their disease [2,3]. The relationship between an oncology patient’s illness and depression has important implications and management problems, as it interferes with the patient’s ability to cope with the disease, decreases treatment acceptance, prolongs hospitalization, increases the risk of suicide and decreases quality of life [4,5]. In addition, psychological distress (depression, anxiety and poor quality of life) has been associated with higher cancer mortality in patients with a history of cancer [6]. Cancer patients have substantially greater levels of depression and anxiety than the overall population, with a prevalence of 20% and 10%, respectively, compared to five percent and seven percent [7,8]. In fact, it has been reported that 30% of oncologic patients suffer from anxiety [9].

Mental illnesses are disabling and, if undetected, can progress in severity and duration [9]. The annual costs for cancer patients are 113% higher when they suffer from depression, producing a significant impact on healthcare systems [10]. In addition, anxiety and depression are undertreated and underappreciated among cancer patients, with mental health care being 41% less attended to in patients who need it [7,11].

Self-perceived health (SPH) [12,13,14] and perceived social support (PSS) [15] are related to high rates of depression in people with cancer or cancer survivors. In Spain, people who have overcome some type of cancer have significantly worse self-perceived health and higher rates of depression than people who have never suffered any type of cancer [16]. PSS—which is understood as the pleasure obtained from getting help from members of a social network [17]—is associated with a higher well-being experience and plays a very significant part in the management of illness and the improvement of health [17,18]. Higher levels of PSS have been shown to be beneficial to the immune system of people with cancer, as people with higher levels of social support live approximately twice as long as those without it [19].

In the general population, poor physical performance predicts the onset of depression in adults [20]. Recent studies have shown the benefits of physical activity for the well-being of people with cancer, demonstrating the usefulness of greater physical activity levels (PAL) and a physical activity index (PAI) as both prevention and therapy for mental illness [21,22]. Thus, a central public health objective is to support the promotion of an active lifestyle based on physical activity (PA) as an important strategy to combat depression [23]. Specifically, in cancer patients, PA and exercise have been shown to be effective in combating cancer-related fatigue [24] and improving quality of life among cancer survivors [25,26]. Oncologic patients that are physically active after their cancer diagnosis have been shown to have a lower risk of cancer recurrence and mortality, as well as fewer serious adverse effects of the disease (including in terms of mood) and disease-related treatment [27,28,29]. Upper levels of PSS have also been related to higher PAL practice at moderate or high intensities [30]. Nevertheless, to the best of our knowledge, no associations have been found between levels of SPH, PSS and PA in people with cancerous tumours.

Therefore, this study aimed to explore the associations between PAL, PSS and SPH, together with mental health and its dimensions in a Spanish adult population with cancerous tumours, which could help to understand how PAL and PSS affected the perceived health and mental health of the Spanish population with tumours in the period prior to the COVID-19 pandemic, allowing for comparison with future post-pandemic data. We hypothesised that higher PAL, PSS and SPH will be associated with fewer signs of mental anguish.

## 2. Materials and Methods

### 2.1. Design

A cross-sectional research study focusing on data from the Spanish Adult National Health Survey 2017 (ENSE 2017) [31], which is the last health data for the Spanish population prior the COVID-19 epidemic, was conducted. The ENSE is a survey performed in Spain at five-yearly intervals, organised by the Ministry of Health, Consumer Affairs and Social Welfare (MSCBS) jointly with the National Statistics Institute (INE). It obtains data about the health condition of the Spanish population. For the ENSE 2017, qualified and certified enumerators conducted these surveys from October 2016 to October 2017.

### 2.2. Participants

A total of 23,089 individuals from 15 to 103 years old and resident in Spain were interviewed for the ENSE 2017. The sample was chosen by means of a stratified sampling system in three randomised stages [31]: first, the municipalities of the strata created were selected, then households were randomly selected, and finally one adult person from each household was selected.

Specifically for this study, only participants who reported having malignant tumours (affirmative response to point p.25.26a “Have you ever suffered from malignant tumours?”) and who answered all questions of the PA (p.113–p.117) and the GHQ-12 (p.47.1–p.47.12), were considered. Additionally, people older than 70 were not included, as they were not questioned regarding PA (5312 individuals). Respondents who answered “No” or “Don’t know/No answer” to point p.25–26a (“Have you ever suffered from malignant tumours?”) were excluded (17,138 participants). One participant was excluded for not submitting all responses to items p.113–p.117 (items on PA performed). Eleven participants were also discarded for not submitting all the answers to items p.47.1–p.47.12 (items corresponding to the GHQ-12). Finally, for analyses that included the PSS variable, participants who did not submit all responses to the corresponding items of the Duke-UNC-11 questionnaire (p130.1–p130.11) were excluded (25 participants). Thus, the total population sample included 627 individuals (235 males and 392 females), who were residents of Spain and aged between 15 and 69 years.

### 2.3. Measures

The variables extracted from the 2017 ENSE for further analysis were:

**Mental health (GHQ-12):** Items 47.1–47.12 were evaluated from the Spanish edition of the Goldberg General Health Questionnaire (GHQ-12) [32]. This is a multidimensional questionnaire that assesses the risk of poor mental health with a score between 0 and 36, with 0 being the lowest risk and 36 being the highest. Each item can be answered with a value between 0 and 3, and a total score of more than 12 is considered to indicate some type of psychological distress [32,33,34]. In the Spanish population this self-report instrument shows a Cronbach α of 0.86. This questionnaire address the following three dimensions [33,34]:

**Successful coping (FI):** Items 47.1, 47.3, 47.4, 47.7, 47.8 and 47.12. With scores between 0 and 18, 0 being the most successful coping and 18 the least. The external validity for this measure has an external validity of 0.82 with a *p*-value of 0.001.

**Self-esteem (FII):** Items 47.6, 47.9, 47.10 and 47.11. With scores between 0 and 12, where 0 is the highest self-esteem and 12 is the lowest. This variable has an external validity of 0.70 and a *p*-value of 0.001.

**Stress (FIII):** Items 47.2, 47.5 and 47.9. With scores between 0 and 9, 0 being the least stressful and 9 the most stressful. This measure shows an external validity of 0.75, with a *p*-value of 0.001.

**Self-perceived health (SPH):** Which is obtained from the responses presented to question 21 (“In the last twelve months, would you say that state of health has been very good, good, fair, poor, bad, very bad?”). In this research we considered SPH as negative for “Fair/Bad/Very bad” responses and positive for “Good/Very Good” responses.

**Perceived social support (PSS):** Items 130.1–130.11 from the Duke-UNC-11 Functional Social Support Questionnaire, whose objective is to evaluate the respondents’ PSS. It is an 11-item questionnaire with five possible answers to each question, with values ranging from 0 (“much less than I want”) to 5 (“as much as I want”) for each of them. The result is obtained from the sum of the responses to all the items, obtaining values between 11 and 55. Values lower than 32 points indicate a low PSS in the Spanish population, the internal consistency of the questionnaire being excellent in this community (α = 0.90) [33,35,36].

**Physical activity index (PAI):** Items 113–116, from the Spanish edition of The International Physical Activity Questionnaire (IPAQ) [37], for moderately and intensely active people. The PAI was adapted using this formula [38]:

*(Intensity factor for vigorous activity × Frequency factor for vigorous activity × Duration factor for vigorous activity) + (Intensity factor for moderate activity × Frequency factor for moderate activity × Duration factor for moderate activity).*

The PAI has values from 0 to 67.5, with 67.5 as the highest PA value [39].

**Physical activity level (PAL):** By combining the PAI with the responses to point 117 of the Spanish edition of the IPAQ (“Now think about the time you have spent walking in the last 7 days”), which has the responses, “Any day more than 10 minutes at a time”, or “From 1 to 7 days more than 10 minutes’ walking”, four PAL groups were established:

- **Inactive:** PAI = 0; they reported not having walked for more than 10 minutes at a time any day of the week.
- **Walker:** PAI = 0; reported walking at least one day a week for more than 10 min.
- **Active:** PAI between 1 and 30.
- **Very active:** PAI greater than 30.

### 2.4. Statistical Analysis

The normality of the data was studied using the Kolmogorov–Smirnov test. A descriptive analysis was achieved, and the sample was characterized by presenting the median values and the interquartile range, complemented by the mean values and standard deviation of the continuous variables (age, mental health, PAI, PSS, self-esteem, stress, successful coping), and the absolute and relative frequencies presented by the categorical variable (PAL and SPH).

The Mann–Whitney U test and Kruskal–Wallis non-parametric statistical tests were performed to analyse possible inter-group or baseline differences for the continuous variables, and the Chi-square test was used to assess possible dependence relationships and differences in proportions among the groups (pairwise z-test for independent ratios) for categorical variables. A Spearman correlation analysis was performed, using the Bonferroni correction, interpreted according to Cohen’s classification (*p* = 0.003).

Multiple binary logistic regression analyses were performed where SPH was taken as the dependent variable and PAL, sex, age, PSS and BMI were taken as independent variables. Thus, linear regressions were performed to predict the variable scores for mental health, successful coping, self-esteem and stress, taking these as dependent variables, and PAL, sex, BMI, age and PSS as independent variables. For all analyses, a level of less than 0.05 was considered statistically significant. IBM SPSS Statistical v.25 software was employed for all the tests in this study.

## 3. Results

The proportion of people who did not perform moderate and/or vigorous PA was over 70% (72.1% in the general population; 74.4% men vs. 70.6% women). Mental health, according to the GHQ-12, presented a median score of 11, with differences between women and men (11 vs. 10. *p* = 0.016). In addition, significant differences were found for self-confidence (*p* = 0.012), with lower scores in men than in women (2 vs. 3), and for stress (*p* = 0.006), although both presented a median score of 3 points (Table 1).

Table 2 shows self-perceived health in people with malignant tumours according to their PAL. Dependence links have been found between PAL and self-perceived health (*p* < 0.001). In addition, the “Active” and “Very active” groups presented differences in the prevalence obtained, being lower in these groups than in the “Inactive” and “Walking” categories (*p* < 0.005).

The mean (and standard deviations) and median (and interquartile ranges) scores obtained by the different PAL groups for mental health and its dimensions are shown in Table 3, based on the GHQ12, both for all participants with malignant tumours and by gender. It was observed that the mean score for mental health decreased as PAL increased in the general population (15.4 vs. 9.0 between the “Inactive” and “Very active” groups), in men (15.7 vs. 7.3) and in women (15.2 vs. 9.7). This decrease in GHQ12 scores as PAL increased, was also found in the medians, with significant differences at baseline, taking PAL as a factor (*p* < 0.001). For the general population and for both genders, similar findings were found for all three dimensions of mental health, with the highest scores in the “Inactive” group (*p* < 0.001) (Table 3).

The links between PAL and mental health and its facets, and the responses to all items of the GHQ-12 are shown in Table 4. Faint inverse links between PAL and mental health were found (rho: −0.274; *p* < 0.001), successful coping (rho: −0.239; *p* < 0.001), self-confidence (rho: −0.264; *p* < 0.001) and stress (rho: −0.239; *p* < 0.001). Weak or very low inverse interactions between PAL and the responses to all questions of the GHQ-12 were also found (Table 4).

Table 5 shows the interrelations between scores on the Duke-UNC-11 and the GHQ-12, showing low inverse links between PSS and mental health (r: −0.225; *p* < 0.001), successful coping (r: −0.218; *p* < 0.001), self-confidence (r: −0.231; *p* < 0.001) and stress (r: −0.165; *p* < 0.001). Furthermore, the weak and very weak inverse correlations found between the PSS scores and the GHQ-12 items are shown in Table 5.

Table 6 shows a multiple binary logistic regression analysis, showing that active and very active people have a smaller risk of poor self-perceived health (Active: Exp(B): 0.342; 95% CI: 0.197–0.595; Very active: Exp(B): 0.182; 95% CI: 0.075–0.444). It was also found that people with a higher PSS had a lower risk of negative self-perceived health (Exp(B): 0.978; 95% CI: 0.955–1.001).

For mental health as a function of sex, age, BMI, PSS and PAL, a result of R^2^ = 11.0% was obtained, positively explained by mental health (constant: β = 22.872, t = 23.874, *p* < 0.001; PAL: β = −1.989, t = −6.475, *p* < 0.001; PSS: β = −0.154, t = −4.787, *p* < 0.001). For successful coping as a function of the same variables, R^2^ = 9.7% was obtained, which was positively explained by successful coping (constant: β = 11.305, t = 17.523, *p* < 0.001; PAL: β = −0.744, t = −6.015, *p* < 0.001; PSS: β = −0.058, t = −4.501, *p* < 0.001). For self-esteem as a function of the same variables we obtained R^2^ = 10.6%, positively explained by self-esteem (constant: β = 8.447, t = 11.396, *p* < 0.001; PAL: β = −0.859, t = −6.046, *p* < 0.001; PSS: β = −0.076, t = −5.074, *p* < 0.001). Finally, regarding stress, as a function of the same variables, R^2^ = 7.2% was obtained, positively explained by stress (constant: β = 6.660, t = 10.824, *p* < 0.001; PAL: β = −0.656, t = −5.561, *p* < 0.001; PSS: β = −0.041, t = −3.329, *p* < 0.001).

## 4. Discussion

The main findings were the associations identified between PAL and GHQ-12, whereby people with tumours who reported a higher PAL presented lower mean scores for the three mental health aspects. Weak associations were also detected between PAL and mental health, successful coping, self-confidence and stress.

The mean scores found in mental health showed that the higher the PAL, the lower the score obtained in the GHQ-12 in people with tumours, which means that increased physical activity translates into better mental health, although the results showed a weak inverse correlation between both variables (r = −0.274).

Cancer patients were found to have an increased risk of mental health problems such as anxiety and psychological distress [40]. Our results show that the effect of physical activity on mental health in patients with malignant tumours may be beneficial. This was examined in two meta-analyses [26,28] in which it was concluded that physical activity has positive effects on psychological outcomes, including a lower prevalence of depression. In the meta-analysis by Cormie et al. [28], 12 articles were examined of which 10 showed significant improvements in one or more psychosocial outcomes. Several articles conclude that engaging in PA, even a small amount at a low intensity, was associated with a significant decrease in mortality [27], a reduction in the rate of self-reported functional impairment [14], and many other benefits [25].

Two articles showing a discrepancy with our results were found [41,42], in which no significant improvements to poor mental health were found when applying movement or physical activity therapies. Newby et al. [41] reported that physical exercise-based interventions showed no statistically significant change in depression scores, but an overall trend of 0.9 units (95% CI: 2.04, 0.25) of benefit was found. These differences could be derive from the type of cancer (prostate), the low intensity of physical activity analysed (e.g., Reiki) or reduced PAL. On the other hand, Bradt et al. [42], reporting on two small-scale trials, found no evidence of an effect on depression, stress, anxiety, fatigue or body image from dance/movement therapy in cancer patients. However, their outcomes do suggest that therapy movement may have beneficial effects on quality of life, but the number of studies is limited. Unlike our research, which did not classify participants according to their PAL, we believe that more active patients may have benefits over less active patients.

In the general population, we found a consensus of results. In younger age groups, children and adolescents, the same trend is observed: regular physical activity is associated with better mental health and well-being [43,44,45]. Bowe et al. [46] reported that positive mental health was reported significantly more frequently in men (20 vs. 12%, *p* < 0.001), whereas negative mental health was significantly more prevalent among women (13 vs. 6%, *p* < 0.001). It has also been shown that those who reported negative mental health were significantly less likely to report being very active (20 vs. 33%, *p* < 0.001). These findings were in line with the results presented in the present article, albeit in the tumour population, with the female population showing a greater tendency to suffer from mental health problems. Dinas et al. [47], reported the positive results of physical activity on acute and chronic depression in comparison with pharmacological antidepressant treatments, thus highlighting the importance of physical activity as a fundamental and applicable tool in society to improve the mental health of the population. Cherubal et al. [48] reported a higher prevalence of mental health disorders in inactive people. On the other hand, Galper et al. [20] state that regular physical activity is transversely associated with less depressive symptomatology and greater emotional well-being; the same is reported in a population with schizophrenia [21], with PA being an effective treatment.

Similar to our results on PAL and mental health, Čuprika et al. [49] reported weak relationships (r = 0.22, *p* < 0.05) between the level of physical activity and the risk of poor mental health in a population of physically active women. This weak relationship is also found between physical activity and the level of self-esteem, shown by Spence and Poon [50].

The present study is a cross-sectional research study for which a correlational analysis was performed, so a cause–effect association between the study variables cannot be established. Furthermore, the quantification of the PAL using indirect tools could be a weakness of the present research, since numerous studies have reported that the IPAQ-SF overvalues the level of PA [51,52]. Therefore, it is recommended to quantify PA in a direct and objective manner to assess its relationship with mental health in the adult population with tumours. As the data were obtained from surveys, response bias may also have had some effect on the results. Finally, the present study did not consider more detailed data, neither on the disease (diagnostic, localization, severity, therapy, modality, duration), nor on other sociodemographic variables that could condition the results, such as marital status, socioeconomic level or educational level; these variables, as well as the heterogeneity of the sample, could have been sources of bias. Future research could explore the effects of these parameters in research that evaluates the connection between PA and mental health in adults with tumours.

## 5. Conclusions

Considering our outcomes, there are weak but significant relationships between PAL, PSS and SPH and mental health in the Spanish population with malignant tumours. Positive SPH prevalence was higher in people with higher PAL and PSS. The inactive population presented worse mental health scores. Thus, physical activity may be an effective complementary treatment for the symptoms of mental illness in cancer patients. In addition, women with cancerous tumours have an increased risk of suffering from poor mental health compared to men. However, more research is needed to study these relationships and to establish effective doses of PA to improve or prevent mental pathologies in people with malignant tumours.

## Figures and Tables

**Table 1:** Descriptive analysis of Spanish adults with cancerous tumours from the ENSE 2017 based on age, self-perceived health, a physical activity index and the dimension-subscales of the GHQ-12, Duke-UNC-11 Perceived Social Support Questionnaire and physical activity level.

Variables	Total n = 627	Men n = 235	Women n = 392	
Age (Years)				^a^
Median (IQR)	59 (13)	60 (13)	59 (14)	0.323
Mean (SD)	57.3 (9.3)	57.6 (9.5)	57.1 (9.2)	
PAI				^a^
Median (IQR)	0 (15)	0 (15)	0 (15)	0.418
Mean (SD)	7.1 (13.8)	6.7 (13.5)	7.3 (14.0)	
Mental health				^a^
Median (IQR)	11 (6)	10 (7)	11 (6)	0.016
Mean (SD)	12.2 (6.0)	11.7 (7.1)	12.6 (6.0)	
Successful coping				^a^
Median (IQR)	6 (1)	6 (1)	6 (1)	0.488
Mean (SD)	6.9 (2.4)	6.9 (2.3)	7.0 (2.5)	
Self-esteem				^a^
Median (IQR)	2 (3)	2 (4)	3 (3)	0.012
Mean (SD)	3.0 (2.8)	2.7 (2.8)	3.2 (2.8)	
Stress				^a^
Median (IQR)	3 (3)	3 (3)	3 (3)	0.006
Mean (SD)	3.3 (2.3)	3.0 (2.4)	3.5 (2.2)	
Perceived social support	Total n = 602	Men n = 220	Women n = 382	^a^
Median (IQR)	50 (9)	49 (9)	50 (9)	0.874
Mean (SD)	47.6 (7.4)	47.5 (7.4)	47.6 (7.4)	
SPH	Total n = 627	Men n = 235	Women n = 392	^b^
Negative	358 (57.1)	141 (60.0)	217 (55.4)	0.256
Positive	269 (42.9)	94 (40.0)	175 (44.6)	
PAL				^b^
Inactive	112 (17.9%)	41 (17.4%)	71 (18.1%)	0.622
Walker	340 (54.2%)	134 (57.0%)	206 (52.6%)	
Active	141 (22.5%)	50 (21.3%)	91 (23.2%)	
Very active	34 (5.4%)	10 (4.3%)	24 (6.1%)	

n, participants; %, percentage; IQR (interquartile range); SD (standard deviation); GHQ-12 (Goldberg’s General Health Questionnaire): scores between 0 and 36, with 0 representing the best mental health and 36 the worst mental health; Successful coping: scores from 0 to 18, with 0 representing the most successful coping and 18 the least successful coping; Self-esteem: scores from 0 to 9, with 0 representing the highest level of self-esteem and 9 the lowest level of self-esteem; Stress: scores from 0 to 9, with 0 representing no stress and 9 very stressed; PAL (physical activity level): Inactive, PAI = 0, the respondent declares they never go for a walk for more than 10 min at a time. Walker, PAI = 0, the respondent reports walking at least one day a week for more than 10 min at a time. Active, PAI = 1–30. Very active, PAI = +30; PAI (physical activity index): scores between 0 and 67.5; ^a^, *p*-value from Mann–Whitney U test); ^b^, *p*-value from chi-square test.

**Table 2:** Self-perceived health in people with malignant tumours, according to their physical activity level.

SPH	Total		Inactive		Walker		Active		Very Active		x^2^	df	*p*	CC
	n	(%)	n	(%)	n	(%)	n	(%)	n	(%)				
Negative	358	(57.1)	81 ^a^	(72.3)	209 ^a^	(61.5)	58 ^b^	(41.1)	10 ^b^	(29.4)	38.6	3	<0.001	0.24
Positive	269	(42.9)	31 ^a^	(27.7)	131 ^a^	(38.5)	83 ^b^	(58.9)	24 ^b^	(70.6)				

SPH (self-perceived health); n (participants); % (percentage); x^2^ (Pearson’s chi-square); df (degree freedom); *p* (*p*-value); CC (contingency coefficient); ^a,b^ (each subscript corresponds to significant differences between column proportions at a 95% z-test for independent proportions. Effect size = 0.26; statistical power (1 − β) = 0.997.

**Table 3:** Links between physical activity level and dimension-subscales of the GHQ-12 in Spanish adults with cancerous tumours from the ENSE 2017.

Variables	Total n = 1661			Men n = 516			Women n = 1145		
**Mental health**									
PAL	m (sd)	mdn (IQR)	*p*	m (sd)	mdn (IQR)	*p*	m (sd)	mdn (IQR)	*p*
Inactive	15.4 (7.7)	12 (11)	<0.001	15.7 (7.9)	13 (12)	<0.001	15.2 (7.6)	12 (10)	<0.001
Walker	12.2 (5.6)	11 (5)		11.5 (5.2)	11 (6)		12.7 (5.7)	11 (6)	
Active	10.6 (4.9)	9 (5)		9.7 (5.1)	8 (6)		11.1 (4.8)	10 (5)	
Very active	9.0 (4.0)	8 (5)		7.3 (2.1)	7 (3)		9.7 (4.4)	9 (6)	
**Successful coping**									
PAL	m (sd)	mdn (IQR)	*p*	m (sd)	mdn (IQR)	*p*	m (sd)	mdn (IQR)	*p*
Inactive	8.3 (3.4)	7 (4)	<0.001	8.3 (3.4)	7 (5)	0.002	8.3 (3.4)	7 (4)	<0.001
Walker	6.8 (2.2)	6 (1)		6.7 (2.0)	6 (1)		6.9 (2.3)	6 (1)	
Active	6.3 (1.5)	6 (0)		6.3 (1.7)	6 (0)		6.3 (1.4)	6 (0)	
Very active	6.0 (1.6)	6 (0)		5.6 (1.0)	6 (0)		6.2 (1.8)	6 (2)	
**Self-esteem**									
PAL	m (sd)	mdn (IQR)	*p*	m (sd)	mdn (IQR)	*p*	m (sd)	mdn (IQR)	*p*
Inactive	4.4 (3.3)	4 (5)	<0.001	4.6 (3.4)	4 (6)	<0.001	4.2 (3.3)	4 (4)	<0.001
Walker	3.0 (2.6)	3 (3)		2.7 (2.5)	2 (3)		3.2 (2.6)	3 (3)	
Active	2.3 (2.6)	1 (4)		1.7 (2.5)	0 (3)		2.6 (2.6)	2 (4)	
Very active	1.6 (2.0)	1 (3)		0.7 (1.3)	0 (1)		1.9 (2.1)	2 (4)	
**Stress**									
PAL	m (sd)	mdn (IQR)	*p*	m (sd)	mdn (IQR)	*p*	m (sd)	mdn (IQR)	*p*
Inactive	4.1 (2.4)	4 (3)	<0.001	4.1 (2.6)	4 (3)	0.001	4.0 (2.4)	4 (3)	<0.001
Walker	3.4 (2.2)	3 (3)		3.1 (2.2)	3 (3)		3.6 (2.1)	3 (3)	
Active	2.7 (2.2)	3 (3)		2.2 (2.4)	2 (3)		3.0 (2.1)	3 (2)	
Very active	1.8 (1.7)	2 (3)		1.1 (1.4)	1 (2)		2.1 (1.8)	2 (3)	

m, mean; sd, standard deviation; mdn, median; IQR (interquartile range); GHQ-12 (Goldberg’s General Health Questionnaire): scores between 0 and 36, with 0 representing the best mental health and 36 the worst mental health; Successful coping: scores from 0 to 18, with 0 representing the most successful coping and 18 the least successful coping; Self-esteem: scores from 0 to 9, with 0 representing the highest level of self-esteem and 9 the lowest level of self-esteem; Stress: scores from 0 to 9, with 0 representing no stress and 9 very stressed; PAL (physical activity level): Inactive, PAI = 0, the respondent declares they never go for a walk for more than 10 min at a time. Walker, PAI = 0, the respondent reports walking at least one day a week for more than 10 min at a time. Active, PAI = 1–30. Very active, PAI = +30; PAI (physical activity index): scores between 0 and 67.5; *p*, *p*-value from Kruskal–Wallis test.

**Table 4:** Correlations between physical activity level and Goldberg General Health Questionnaire responses in Spanish adults with cancerous tumours.

Target Variable	rho	*p*
Mental health	−0.274	<0.001
Successful coping	−0.239	<0.001
Self-esteem	−0.264	<0.001
Stress	−0.239	<0.001
1. Have you been able to concentrate well on what you were doing?	−0.120	<0.001
2. Have your worries caused you to lose sleep?	−0.161	0.002
3. Did you feel that you were playing a useful role in life?	−0.198	<0.001
4. Did you feel able to make decisions?	−0.205	<0.001
5. Have you felt constantly overwhelmed and under stress?	−0.191	<0.001
6. Have you had the feeling that you cannot overcome your difficulties?	−0.262	<0.001
7. Have you been able to enjoy your normal daily activities?	−0.211	<0.001
8. Have you been able to cope adequately with your problems?	−0.221	<0.001
9. Have you felt unhappy or depressed?	−0.250	<0.001
10. Have you lost confidence in yourself?	−0.167	<0.001
11. Have you thought of yourself as a worthless person?	−0.184	<0.001
12. Do you feel reasonably happy considering all the circumstances?	−0.199	<0.001

GHQ-12 (Goldberg’s General Health Questionnaire): scores between 0 and 36, with 0 representing the best mental health and 36 the worst mental health; Successful coping: scores from 0 to 18, with 0 representing the most successful coping and 18 the least successful coping; Self-esteem: scores from 0 to 9, with 0 representing the highest level of self-esteem and 9 the lowest level of self-esteem; Stress: scores from 0 to 9, with 0 representing no stress and 9 very stressed; PAL (physical activity level): Inactive, PAI = 0, the respondent declares they never go for a walk for more than 10 minutes at a time. Walker, PAI = 0, the respondent reports walking at least one day a week for more than 10 minutes at a time. Active, PAI = 1–30. Very active, PAI = +30; PAI (physical activity index): scores between 0 and 67.5; rho, Spearman’s correlation coefficients with the Bonferroni correction factor having *p* = 0.003; *p*, *p*-value.

**Table 5:** Correlations between perceived social support and Goldberg General Health Questionnaire responses in Spanish adults with cancerous tumours.

Target Variable	Correlations	*p*
Mental health	−0.127	<0.001
Successful coping	−0.133	<0.001
Self-esteem	−0.152	<0.001
Stress	−0.117	<0.001
1. Have you been able to concentrate well on what you were doing?	−0.095	0.020
2. Have your worries caused you to lose sleep?	−0.054	0.183
3. Did you feel that you were playing a useful role in life?	−0.083	0.042
4. Did you feel able to make decisions?	−0.116	0.004
5. Have you felt constantly overwhelmed and under stress?	−0.091	0.026
6. Have you had the feeling that you cannot overcome your difficulties?	−0.116	0.004
7. Have you been able to enjoy your normal daily activities?	−0.137	0.001
8. Have you been able to cope adequately with your problems?	−0.133	0.001
9. Have you felt unhappy or depressed?	−0.162	<0.001
10. Have you lost confidence in yourself?	−0.132	0.001
11. Have you thought of yourself as a worthless person?	−0.146	<0.001
12. Do you feel reasonably happy considering all the circumstances?	−0.123	0.002

GHQ-12 (Goldberg’s General Health Questionnaire): scores between 0 and 36, with 0 representing the best mental health and 36 the worst mental health; Successful coping: scores from 0 to 18, with 0 representing the most successful coping and 18 the least successful coping; Self-esteem: scores from 0 to 9, with 0 representing the highest level of self-esteem and 9 the lowest level of self-esteem; Stress: scores from 0 to 9, with 0 representing no stress and 9 very stressed; Functional Social Support Questionnaire: scores between 11 and 55 points; Spearman’s correlation coefficients with the Bonferroni correction factor having *p* = 0.003; *p*, *p*-value.

**Table 6:** Multiple binary logistic regression analysis for the self-perceived health negative risk factor.

	B	S.E.	Wald	df	Sig.	Exp(B)	95% CI for EXP(B)	
							Lower	Upper
Inactive			24.302	3	0.000			
Walker	−0.427	0.247	2.991	1	0.084	0.652	0.402	1.059
Active	−1.072	0.281	14.499	1	0.000	0.342	0.197	0.595
Very active	−1.702	0.454	14.043	1	0.000	0.182	0.075	0.444
Sex (Men)	0.111	0.180	0.376	1	0.540	1.117	0.784	1.591
PSS	−0.022	0.012	3.435	1	0.064	0.978	0.955	1.001
BMI	0.031	0.020	2.559	1	0.110	1.032	0.993	1.073
Age	0.021	0.009	5.280	1	0.022	1.022	1.003	1.041
Constant	−0.187	0.878	0.046	1	0.831	0.829		

B, under-standardised beta; SE, standard error of regression; Wald, Wald Chi-square test; Df, degree freedom; Sig., statistical significance; Exp, exponential regression; CI, confidence interval; PSS, perceived social support; BMI, body mass index.

## Data Availability

The data used were obtained from public use files, which are available on the website of the Spanish Ministry of Health, Consumer Affairs, and Social Welfare: https://www.mscbs.gob.es/estadEstudios/estadisticas/encuestaNacional/encuesta2017.htm (accessed on 20 October 2022).
